# Radiation therapy considerations during the COVID‐19 Pandemic: Literature review and expert opinions

**DOI:** 10.1002/acm2.12898

**Published:** 2020-05-14

**Authors:** Pranshu Mohindra, Courtney R. Buckey, Shifeng Chen, Terence T. Sio, Yi Rong

**Affiliations:** ^1^ Department of Radiation Oncology University of Maryland School of Medicine Baltimore MD USA; ^2^ Department of Radiation Oncology Mayo Clinic Arizona Phoenix AZ USA; ^3^ Department of Radiation Oncology University of California‐Davis Cancer Center Sacramento CA USA

**Keywords:** coronavirus, COVID-19, quality assurance, radiotherapy, World Health Organization

## 1 Introduction

Coronavirus disease 2019 (COVID‐19) is an unprecedented pandemic that has already reached over 2 million confirmed cases globally, with at least 140,000 deaths as reported by the World Health Organization (WHO) as of April 16, 2020.^1^ More than 662,000 cases have been reported in the United States with more than 29,000 deaths.^2^ The overall crude mortality rate now stands at 6.6% (may possibly be lower due to under‐testing and under‐reporting of total confirmed cases), and is highly dependent on age group, comorbidities, and the locoregional resources medically.^1^ A report from the United States presented age‐stratified COVID‐19‐associated hospitalization rates among 1,482 patients during March 1–28, 2020, highlighting an alarmingly high rate of 74.5% at age > 50 years with underlining medical conditions.^3^ Based on a data summary report provided by New York City Health, as of April 14, 2020, the shares of a total of 6839 deaths reached 0.04%, 4.5%, 23.1%, 24.6%, and 47.7% for the age groups of 0–17, 18–44, 45–64, 65–74, and 75+ years old.^4^ All data suggest that adults at a more advanced age group are facing higher morbidity and mortality risks.

Clearly, with our aging population, cancer patients are among this most vulnerable group, which brings us to this editorial regarding special considerations for radiotherapy (RT) during a COVID‐19 pandemic. The global COVID‐19 paradigm is ever‐changing, thus this discussion is based on our current situation as of April 16, 2020, including increased risks of COVID‐19 exposure to healthcare workers,^5^ significant shortage of personal protective equipment (PPE)^6^ for healthcare workers, severely limited testing capacity only available for symptomatic patients,^7^ therapeutic drugs still being at experimental stage,^8^ and the prospect of vaccinations at least a year away (still under development),^9^ etc. Questions raised among our RT community include “Can quality‐assured RT treatment be safely provided to COVID‐19 positive patients?,” “How to best protect other cancer patients and staff from being infected?,” “What if patients are confirmed positive mid‐way through the RT treatment,” etc. In fact, a very recent case report from M.D. Anderson Cancer Center just revealed an asymptomatic nonsmall cell lung cancer patient who passed regular COVID‐19 screening but demonstrated internal development of multifocal ground glass opacities on the thoracic CT‐on‐rail scan prior to the first fraction of RT treatment. The patient was then subsequently confirmed COVID‐19 positive.^10^ To treat, or not to treat? There will not be a simple answer to the question. Herein, we have Dr. Pranshu Mohindra and Dr. Shifeng Chen from University of Maryland, Baltimore elaborating their proposition on “*Quality assured radiotherapy can be delivered during COVID‐19 pandemic,*” and Dr. Courtney Buckey and Dr. Terence Sio from Mayo Clinic Arizona offering their opinions on “*Radiotherapy treatment should be postponed for COVID‐19 positive patients.*”

Pranshu Mohindra, MD, MBBS is a radiation oncologist and an Associate Professor of Radiation Oncology at the University of Maryland School of Medicine, Baltimore. His primary area of clinical and research interest is in thoracic/lung, gynecological, and hemato‐lymphoid malignancies using both modern photon and proton‐based radiotherapy approaches. Relevant to this editorial, Dr. Mohindra leads the Scope of Practice, Policy and Procedure Management Workgroup of Quality and Safety Review Council in his department and also serves as Director of Billing and Compliance. In these roles, he oversees the development of Standard Operating Procedures for various departmental clinical activities and establishes uniform procedures system‐wise, along with overall clinical billing and compliance.

Courtney R Buckey, PhD, DABR is a medical physicist and Assistant Professor of Radiation Oncology at the Mayo Clinic in Arizona. Related to this editorial, her focuses are patient and staff safety, event reporting, introducing techniques like Humble Inquiry and the London Protocol to local incident investigation, and teaching quality and safety to physics residents. Dr. Buckey’s current AAPM participation includes serving as vice‐chair of Task Group 314‐Guidance for Fault Recovery in Radiation Therapy; a member of the Working Group for the Prevention of Errors, and the Women’s Professional Subcommittee; and a therapy track organizer on the Spring Clinical Meeting Subcommittee. She is currently the president‐elect of the Arizona Chapter of the AAPM.

Shifeng Chen, PhD is an Associate Professor, Associate Chief of Clinical Physics, and Director of the Dosimetry Training Program, in the Department of Radiation Oncology at the University of Maryland School of Medicine. He received his BS in Engineering Physics from Tsinghua University at Beijing, China, and a PhD degree of Physics from Florida State University. He then completed postdoctoral training in Radiation Oncology Physics at Duke University Medical Center. His clinical and research interests include Treatment Planning, GRID/LATTICE Radiation Therapy, Stereotactic Radiation Therapy, Thermal Therapy, Automation for Radiation Oncology, and Machine Learning. He has authored and coauthored 79 peer‐reviewed journal articles, 2 book chapters, and 82 conference abstracts.

Terence T. Sio, MD, MS is a radiation oncologist and Associate Professor of Radiation Oncology who specializes in both photon‐based and proton radiotherapies. His main focuses are in gastrointestinal, lung, and brain cancers including brain metastasis treatments using stereotactic radiosurgery. He has a strong interest in ion beam radiotherapies internationally.

## 2 Quality assured radiotherapy can be delivered during COVID‐19 pandemic — Pranshu Mohindra, MD, MBBS and Shifeng Chen, PhD

With the overwhelming and fast‐increasing numbers of COVID‐19, expectedly, the availability of healthcare resources has been significantly compromised.^11^, ^12^, ^13^ Radiation oncology departments are not immune to these challenges especially from resource limitation such as PPE and risk/fear of staff exposure impacting day‐to‐day practices. Hence, the feasibility of delivering quality assured RT during the pandemic has been called into question leading to the theme of this debate. Through a series of arguments below, we emphasize that cancer care cannot stop and RT is integral to cancer care during the pandemic which is unfortunately expected to last for an extended duration. As such emphasis of institutional policies should be to establish stringent infection control measures to safely treat both COVID‐19 positive and negative cancer patients, while ensuring safety of staff and providers.

### 2.A Cancer care cannot stop

While the pandemic has clearly impacted the world in more ways than one could have possibly imagined, a demon that cannot be ignored is cancer. Per WHO data, cancer resulted in an estimated 9.6 million deaths in the year 2018 accounting for 1 in 6 deaths.^14^ This translates to 800,000 deaths per month, a number much higher than the current pandemic. The stake is high for the cancer patients who by virtue of their immunocompromised state are at a higher risk of contracting and suffering serious complications from COVID‐19 infection.^15^, ^16^, ^17^, ^18^, ^19^ At the same time, patients have an equally challenging fight at hands with the cancer itself which in many cases may be a more urgent problem. Untreated high‐grade brain tumors, advanced head and neck (HN), lung, esophageal, hepato‐biliary‐pancreatic, and hemato‐lymphoid malignancies can result in death in a period of few months or with severe symptomatic progression clinically. These patients may not have the luxury to wait 2–4 months, which is the period that may be needed for a nation to move past the COVID‐19 surge activity with subsequent reduction in patient burden. History supports this need to continue cancer care without interruption. In an analysis of the National Cancer Database for impact of hurricane disaster, the longer radiation treatment duration in patients affected by a hurricane disaster (66.9 vs. 46.2 days; *P* < 0.001) was correlated with significantly worse overall survival.^20^ The adjusted relative risk for death increased with increasing length of the disaster declaration. The fact is that cancer care must simply go on uninterrupted!

### 2.B Radiotherapy — even more important during pandemic

Furthermore, many oncological guidelines are recommending changes in practices for systemic therapy and surgery to minimize immune‐compromising effects or adding comorbidities which could predispose to serious complications during the pandemic.^21^ In a nationwide analysis in China, undergoing chemotherapy or surgery was correlated with a higher risk of clinically severe COVID‐19 events in cancer patients than not receiving chemotherapy or surgery (75% vs 43%, *P* = 0·0026).^16^ Incidentally, the one treatment modality which can be safely delivered with relatively less impact on systemic immune system is radiotherapy. With nearly 50–60% patients with cancer ultimately needing RT in their lifetime,^22^, ^23^ in the setting of deferred chemotherapy and surgery, RT may play an even more important role in management of many cancers.

### 2.C Infection control measures in radiotherapy clinics

WHO has issued key considerations for occupational safety and health and infection prevention and control for health workers during the pandemic.^24^, ^25^ The need to use PPE for RT delivery is not a novel challenge in radiation oncology clinics where patients needing contact precautions are routinely treated, especially in clinics attached to in‐patient units. If the proper infection control measures and safety workflow are developed and implemented, the RT can still be delivered safely with less infection risk of the patients and staff members. At Hubei Cancer Hospital in Wuhan, multiple factors, including the use of incremental infection control zones established in the radiation oncology clinic with appropriate levels of protection, education of staff and patients on infection control, updated cleaning and disinfection policies, daily symptom testing, deployment of special staff rotating schedules, and appropriate waste disposal, allowed uninterrupted RT care during the worst COVID‐19 outbreak period without any known infection to patients or staff members.^26^ Similar experiences were demonstrated during the Severe Acute Respiratory Syndrome epidemics.^27^ As such, in response to the current pandemic, a number of guidelines are now published providing recommendations for practice changes to optimize use of resources and maximizing patient and personnel safety in radiation oncology clinics.^28^, ^29^, ^30^, ^31^, ^32^ Major institutions have released operational guidance regarding practice of RT during the pandemic.^33^, ^34^, ^35^, ^36^, ^37^, ^38^, ^39^ It is proposed that use of appropriate PPE both by patients and RT personnel along with effective patient/staff screening at the entrance point to the clinic will help reduce spread of the infection between asymptomatic patients and staff.

### 2.D Quality assured radiotherapy during pandemic

While the pandemic is not expected to have significant impact on a clinic’s ability to complete pretreatment patient‐specific quality assurance (QA) and machine QA, the need to follow stringent infection precautions coupled with staff anxiety of possible infection risk are likely the biggest impediments in delivery of quality assured RT during the period of pandemic. Fogged eye‐glasses, choking face masks, inconvenient jump suits, and tendency to minimize time of contact with patients are all ingredients for possible personnel errors and decreased focus for attention. From the perspective of being able to deliver quality assured RT, teams need to take multiple steps to minimize the staff member’s exposure. Planning measures such as minimizing beam angles with couch kicks, limiting use of beam accessories like wedges that require close proximity to the gantry and patients, avoiding use of gated‐breath hold techniques which require extensive therapist’s effort or avoiding rectal balloons for prostate treatments, will reduce therapist’s exposure and time of contact with the patients. QA measures such as obtaining portal images without patients in the room for 3DCRT treatments, performing gantry collision assessments prior to patient setup on couch, optimal use of faster orthogonal KV imaging in preference to daily CBCT will reduce time needed for patients to be in the treatment rooms. Scheduling all machines’ and patient‐specific QA over the weekends will reduce the physicist’s exposure. These measures combined with ensuring more frequent breaks for staff using a rotating schedule and psychological support measures will help reduce staff fatigue and possibilities of error.

### 2.E Logistic and ethical issues of a restrictive policy

Pursing a policy that is restrictive in choosing patients who can or cannot receive RT during the pandemic generates multiple logistic and ethical dilemmas. Anecdotal experiences have described patients attempting to hide their symptom or travel history in order to avoid the interruption or delay of their treatments. Such patients could put staff members and other patients at even higher level of danger for infection. By pursuing an open policy that allows continued treatment of all cancer patients during the COVID‐19 outbreak may not only improve patient engagement in the infection control measures but also allow patients a chance to receive optimal cancer care.

Downstream impact of current delays also need to be accounted for. It is plausible that some of the patients for whom treatment is delayed develop cancer progression in the interim, thereby requiring more intensive therapies or use of additional treatment modalities in future. Not only does this create an ethical dilemma in minds of patients and providers when taking a decision about treatment delay, it has legal ramifications with potential for malpractice claims against clinics and providers. This risk of missed‐cancer events is also high since routine follow‐up imaging and clinic visits are also being delayed. Finally, deferring treatments now is likely to result in a surge of cancer patients who need treatment in the postpandemic time, which will generate additional logistic issues and further delays to accommodate the surge.

### 2.F Radiotherapy care for influenza‐like illness/persons under investigation/COVID‐19+ patients

The challenge is exaggerated for patients who are designated to have influenza‐like illness (ILI), persons under investigation (PUI) who have been tested for COVID‐19 with results pending, and actual COVID‐19 positive (COVID‐19+) patients. Many primary care disciplines are continuing to engage with COVID‐19 patients and in fact are playing a key role in early detection of such patients so appropriate quarantine precautions in close contacts can be initiated. Emergency departments and hospital intensive care units in many “hot‐spot” locations have seen an increase in the number of COVID‐19 patients who need extensive care. In times like these, should radiation oncology departments truly distance themselves from providing care to patients for whom in other situations we debate with our other oncology colleagues regarding the strong merit in offering RT? This is even more relevant in this pandemic where a vast majority of the patients can be infectious while being entirely asymptomatic.^40^ Hence, outside of universal testing, there is no feasible way to accurately assess the prevalence of infection in general population which can continue to transmit infection. Does that then justify pursuing a discriminatory approach for the patients who have been tested positive?

It is important to note that while we support treatment of all patients, we recognize that some triage measure during the COVID‐19 pandemic is necessary guided by each local institution/region’s burden of the pandemic. It is evident that radiation oncology is a discipline of interdisciplinary care with multiple members working together to deliver safe RT for our patients. Should a clinic be in a situation where staff availability is significantly crippled, then it will be imperative to initiate cutbacks by delaying initiation of therapy for new patients even if asymptomatic from COVID‐19 or allowing a short duration of interruption in therapy for lower‐risk oncological indications. This would allow channeling of personnel and PPE resources to treat a relatively smaller number of patients with higher‐risk oncological indications. Use of hypofractionated regimens is another such approach to deliver effective RT without compromise with the purpose of limiting the number of visits needed. Ultimately each institution has to make a decision in the best interest of their patients and personnel under their unique circumstances and guided by its local policies.

A step further into the debate relates to the hypothesis of use of low‐dose whole‐thoracic RT as a means to limit and possibly reverse the acute respiratory distress syndrome (ARDS) that is associated with COVID‐19.^41^, ^42^, ^43^, ^44^, ^45^ Clinical protocols are in development to test this hypothesis in COVID‐19 patients and there is a real possibility that some departments may treat noncancerous COVID‐19 patients! Experience of treating COVID‐19+ cancer patients may just be the step in that direction.

## 3 Radiotherapy treatment should be postponed for COVID‐19 positive patients — Courtney R. Buckey, PhD and Terence T. Sio, MD, MS

It is never easy to tell a patient that they have a life‐threatening disease. It is even harder to tell a patient they have two, distinct, life‐threatening diseases. It strains credulity that we should tell them that until they are COVID‐19 negative, that we will not treat their cancer with radiation — but indeed that is what we must strongly consider. During this public health emergency, for the safety of the staff, COVID‐19 negative patients, *and* COVID‐19 positive patients (we do not yet know how delaying radiotherapy will affect the course of their disease process individually, vs. the risks compounded by the COVID‐19 pandemic), we need to strongly consider suspending their RT courses under treatment, and not begin new courses, for COVID‐19 positive patients.

Owing to multiple factors including immunocompromised status, frequent use of hospital and medical facilities, needs for sustained contact with their healthcare givers and providers, cancer patients are more likely to be infected or coinfected by COVID‐19, and, more critically, compared to the general population, they are at a much higher risk of having a severe event (ICU admission, intubation, or death; 39% vs. 8%, *P* < 0.001).^16^ In fact, the most important factor leading to morbidity, as one Chinese study mentioned,^46^ is known exposure to an infectious source (regardless of cancer type). Similarly, the Italian data also pointed that older age, cancer, and past smoking history were risk factors for death; 20.3% of their COVID‐19 deaths were with active cancer.^47^, ^48^ In the absence of any proven vaccine or medication, prevention and mitigation are our *only* weapons in slowing down the spread of the coronavirus, and consequently preventing and averting death for *everyone* in the community including our cancer patients and also their healthcare givers. In early April, COVID‐19 already overtook “cancer” and also “heart disease” (1,641 and 1,774 persons daily on average, respectively), and became the #1 cause of death (reaching even more than 2,000 persons daily on selected days) in our country.^49^ As a result, a significantly conservative approach should be strongly employed and evaluated by each medical institution, in evaluating *every* cancer patient who will need to be evaluated for a course of radiotherapy; we may not be able to afford a “cookie‐cutter,” one policy‐for‐all approach in the face of this unusual pandemic globally. For each individual patient, considerable time should be spent in discussing whether the treatments can be safely postponed by 1–2 months (e.g., a patient with a resected craniopharyngioma), or if alternative therapy (initiating hormonal therapy or active surveillance now for prostate cancer), or a different schedule of radiotherapy (SBRT or hypofractionated treatments) can be used, *regardless* of their COVID‐19 status. The postponing or shortening of the anticipated RT treatments is a balance of benefits and risks from possible cancer progression, vs. the increased risks and safety burdens for both our patients and also staff ourselves.

The Spanish College of Nursing suggested that up to one third of all nurses in Spain, or 70,000 of them, may have been infected by COVID‐19; 30% of the surveyed nurses mentioned that they had symptoms^50^; a CDC report^5^ documented that over 9,000 healthcare providers were already infected by COVID‐19, which was 19% of the data whereas the healthcare worker status was also reported — this would be a *highly* undesirable situation for all of our RT staff and patients if this were the percentages and statistics for our departments or cancer centers; for some centers, that may mean their operations would come to a halt altogether, and they would not be able to treat *any* patient at all. The default radiotherapy workflow for a patient with suspected or confirmed COVID‐19 should be cancelation or delay of their entire radiotherapy treatment course; only emergency indications (uncontrolled brain metastases, severe bleeding, and cord compression, as examples) should be considered for treatment. For suspected patients, the testing results are now coming back faster in most regions of the United States (typically within 48 hr), so the decision‐making process should be faster compared to where we were at the start of this pandemic (circa early March 2020). Confirmed patients may resume their cancer treatments once they have become asymptomatic, complete a 14‐day quarantine, and ideally with two (2) negative COVID‐19 tests that are at least 24 hr apart (subject to change pending new research and guidelines, for example, with emerging ways of testing for serologic evidence of SARS‐CoV‐2‐related antibodies that are increasingly more prevalent now); further guidelines that are more appropriate for our own specialty will also need to be developed in the future.

For patients with confirmed COVID‐19 infections, a detailed laboratory‐based study^51^ showed that viral shedding peaked on or 2–3 days before the onset of symptoms, and the viral load gradually declined as the patient became more symptomatic and sicker; a small period of RT delay, even for 1–2 weeks, can go a long way in decreasing the infection risks to others. For the patient, in most situations, delayed or interrupted radiotherapy treatments can still be more beneficial, especially considering that the patient may be symptomatic or having life‐threatening respiratory issues by COVID‐19, in addition to the side effects that radiotherapy may bring (consider “nonmaleficence” as a medical ethics issue). The safety and increased risks of infections toward *other* patients and staff by needlessly having a COVID‐19 patient in the radiotherapy department must also be considered (consider “parity,” “distributive justice,” and “social justice” as medical ethic principles). The burden of justification, as mentioned above, is very high for deciding COVID‐19+ patients should be routinely treated with radiotherapy. In an actively deteriorating COVID‐19 patient, radiotherapy should be withheld (consider if “beneficence” will become negligible or absent, or if continuing RT may even become harmful); 70‐80% of patients who end up on a ventilator due to COVID‐19 will eventually die as a result based on current reports.

There will be much written about the pandemic, its impact on radiotherapy, and what courses of action are best. But at the most local level, every department will have to wrestle with the following: Can we continue treating all patients, including those that are COVID‐19 positive? How would we do it? Can it be safely completed for staff and patients? Here are the possible scenarios:

### 3.A Continue treatment of COVID‐19 positive patients, with scheduling and treatment unchanged

This scenario probably has the highest risk to the other patients in the cancer center. There is no limit on the number of COVID‐19 positive patients on treatment, and no sequestration from the normal daily schedule. Wait times between patients would become excessive, and schedule disruptions would abound. The CDC’s infection control design goal for an x‐ray treatment room is 6 air changes per hour, and at that rate it takes 46 min to reach 99% efficiency at removal of airborne contaminants — and 69 min for 99.9% efficiency.^52^ That means any routine 15‐min treatment would stretch to fill at least an hour, plus completion of required intense cleaning for the multitudes of surfaces and intricate areas of the immobilization devices and treatment machinery. Maybe a facility thinks it can handle “just one” patient, but then there appears a second, and a third…how many is too many, and when should one draw a line? Even if a single treatment room became the “COVID vault” we might still need to consider the compounded risks of cross‐contamination issues among the COVID‐19 positive patients, as more than one strain of mutations likely already exist in North America.^53^

### 3.B Continue treatment of COVID‐19 positive patients, but with shorter courses, and higher per fraction doses

If this shorter course and/or higher dose scheme were the best course of action, one hopes it would have already been prescribed. This plan does not remove the risk to other patients or staff, as the COVID‐19 positive patients would still be on treatment daily. Short courses for palliation have not been universally adopted, and there is an article in press which shares strategizes for triaging and shortening radiation therapy for oncologic emergencies in an epicenter of COVID‐19 in the United States.^29^ A minor consideration when changing a shorter course/higher dose scheme would be a potential increase in toxicity rates (depending on disease site). A much larger risk would be moving to schemes which have not been clinically validated or performed in a particular radiotherapy center, for example, suddenly changing course to favor an SBRT regimen without the appropriate program in place for QA, margins, planning, constraints, immobilization, and imaging.

### 3.C Continue treatment of COVID‐19 positive patients, with unchanged fractionation patterns, but outside of normal hours

For multiple reasons, this plan is probably the riskiest for the staff. Radiation therapists, already worn thin by the stress of living normal lives during a pandemic, are now likely operating in departments which are understaffed owing to prophylactic reduction in the workforce, or even actual staff sicknesses. Add to that having to now treat patients every day during unusual hours, with a concomitant lack of typical physics, physician, and engineering support, and the stage is set for a higher chance of blunders. Working while tired and out of routine is questionably sustainable and likely to cause or propagate more errors. Treating some number of COVID‐19 positive patients every night is not akin to a normal on‐call situation, where emergencies are rare and staff are often more fresh in attention and energy.

### 3.D Continue treatment of COVID‐19 positive patients, but only at designated centers per city or region

This plan would require an unprecedented amount of coordination. Could we designate and safely staff enough of these centers, in a short enough time frame to be helpful? And in terms of patients getting to the treatment location, there are so many considerations: Is it close enough to population centers? Would we really want COVID‐19 positive patients to leave their homes, potentially with an increased use of public or private transit? What will happen when they are so ill they become inpatients, either from their cancer, comorbidities, or COVID‐19? Would an outpatient department be willing to treat exclusively positive patients? If we did cluster these patients into selected centers, the previously voiced concerns about air exchanges and cleaning/sanitization come ever more into focus. All of the above is based on the idea that centers can and would be able to accept patients mid‐treatment, from centers with disparate technology, dissimilar techniques, and unique workflows. That is not at all a certainty or possibility in a majority of metropolitan cities in America.

### 3.E Continue treatment of selected COVID‐19 positive patients, where morbidity/mortality would be beyond a specified metric

The challenge here lies with the selection criteria. How would it be accomplished? The authors are unaware of codified guidance at this time, meaning that a case‐by‐case, or locally developed metric would be needed. The responsibility/liability/emotional baggage of establishing policies is a tremendous one to shoulder alone. Determining which cases can truly be avoided, delayed, deferred, shortened, or otherwise paused is difficult. Only emergencies? Only palliation? Only curative? Only if so many fractions were already complete? To emphasize this final rhetorical question, the idea of stopping a course of treatment mid‐stream is even more logistically and emotionally challenging than simply never starting. Viable alternative pathways and the ability to defer radiotherapy may evaporate incrementally with which each successfully delivered fraction. We know that under ideal conditions, the guiding principle is to “exert all efforts to retain the planned irradiation schedule” to avoid accelerated repopulation, but in all practicality we are pessimistic that it can be fully executed and accomplished.^54^

The RT treatment delivery team is complex, specialized, highly trained, strictly regulated, and not easy to replace. The loss of any staff member to sickness may be unavoidable now that community transmission is more prevalent (and inevitable), but introducing the known COVID‐19+ patient into the department puts everyone at additional risk. Physicians from other disciplines cannot simply take a refresher course and become a clinically competent radiation oncologist. Physicists from high energy laboratories cannot review a few online presentations and safely QA the treatment plans and treatment machines needed for radiation delivery. Routinely, there is no spare pool of radiation therapists at one’s center to upskill or repurpose. As of the time of publication, the authors are not aware of any emergency purviews for credentialing radiation workers, including physicists and physicians, from outside of our specialty; this is a different situation for letting family physicians or surgeons temporarily run intensive care units due to staff shortage. As a result, over a number of highly hypothesized scenarios, we can see that the burden of treating COVID+ patients can be high; in fact, it may easily overcome the benefit‐to‐risk ratio which, of course, is well‐intended by all parties. The leadership for each individual radiotherapy center must make highly individualized decisions that are suitable for their own needs and beliefs on this controversial issue.
